# Molecular Characterization of Sexual Diversity in a Population of *Serpula lacrymans*, a Tetrapolar Basidiomycete

**DOI:** 10.1534/g3.112.003731

**Published:** 2013-02-01

**Authors:** Inger Skrede, Sundy Maurice, Håvard Kauserud

**Affiliations:** * Microbial Evolution Research Group (MERG), Department of Biology, University of Oslo, N-0316 Oslo, Norway; † Université de Brest, UEB, Laboratoire Universitaire de Biodiversité et Ecologie Microbienne, 29280 Plouzané, France

**Keywords:** mating type, tetrapolar, fungus, dry rot, population diversity

## Abstract

Different mating systems have evolved in the fungal kingdom, including a tetrapolar multiallelic mating system in many basidiomycetes. In tetrapolar species, the presence of different alleles at two mating loci (MAT A and MAT B) is necessary for mating to occur. The tetrapolar fungus *Serpula lacrymans* causes wood-decay in buildings in temperate regions worldwide and is present in Europe with a genetically homogeneous founder population. Using genome sequence data, we annotated the two mating type loci for *S*. *lacrymans* and found the expected synteny with other basidiomycetes, except for a retrotransposon being present in one locus (MAT A). We developed markers linked to the MAT A and MAT B regions and used these to investigate the mating type diversity in the European population. Moreover, we found a good match between the genetic markers and functional mating types as revealed by segregation and mating studies. A low diversity of mating types is present in the European *S. lacrymans* population caused by the founder event where a limited number of genotypes were introduced. This finding contrasts the situation in natural fungal populations where a high diversity of mating types is normally present. Although *S. lacrymans* has a large and viable population in Europe, we argue that the low mating type diversity restrains the dispersal and establishment of the fungus.

In fungi, there are a diversity of mating systems regulating sexual activity and recombination. Functionally, the mating system in ascomycetes resembles what we find for animals with two mating types, whereas most basidiomycetes possess a more intricate system with multiple mating types. The mating type can be considered the sex of a fungus; for two fungi to mate, they need to have different mating types. Basidiomycetes have either a bipolar or a tetrapolar mating system. The bipolar mating system is defined by one mating type locus with two or more alleles, whereas a tetrapolar basidiomycete have two mating type loci, termed MAT A and MAT B, where different alleles at both loci are needed for a mating event to occur. MAT A encodes homeodomain transcription factors, whereas MAT B encodes pheromones and pheromone receptors. Together, they control mate recognition, clamp connection formation, and migration and pairing of nuclei in the formation of dikaryotic mycelium (Brown & Casselton 2001; Heitman *et al.* 2007).

The MAT loci of several basidiomycetes have been characterized (see Heitman *et al.* 2007). Most contain two divergently transcribed homeodomain transcription factors, homeodomain 1 transcription factor, or HD1, and homeodomain 2 transcription factor, or HD2 (James 2007; Kües *et al.* 1994). For a successful mating to happen, the HD1 gene product from one strain must interact with the product of the HD2 gene from another strain. In the Agaricomycetes, the gene order of the MAT A locus and adjacent linked genes are highly conserved (James 2007). More specifically, in all investigated species of the Agaricomycetes (except *Schizopyllum commune*) the two HD transcription factors are flanked by the genes mitochondrial intermediate peptidase (*mip*) and the beta-flanking protein (bfg) (James *et al.* 2004a; Kües *et al.* 2001; Kües *et al.* 2011; Ohm *et al.* 2010). However, alleles of HD transcription factor genes often are highly divergent even if the gene order is conserved. Thus, despite that the gene order of the flanking region of the MAT A locus is conserved, little evidence exists of reduced recombination in the mating type genes per see (Larraya *et al.* 2000; Lukens *et al.* 1996). Recombination may actually be promoted between the HD genes, which could explain previously identified linkage map distances of 16 cM for the genomic region containing MAT A (Raper 1966).

The gene order of the MAT B locus is not as conserved as MAT A (Niculita-Hirzel *et al.* 2008). Nevertheless, the locus seems to consist of genes encoding G protein−coupled 7-transmembrane pheromone receptor genes (termed STE3-like pheromone receptor genes) and their corresponding pheromone precursor genes. For successful mating, the pheromone produced by a pheromone precursor gene in one strain must react with a pheromone receptor in another strain.

In populations of both bi- and tetrapolar basidiomycetes, a high number of MAT alleles typically occur (Heitman *et al.* 2007; Kües *et al.* 2011). Because of the high diversity of MAT A and MAT B alleles, it has proven difficult to sequence and analyze these genes for large populations. Instead the known conserved gene organization has been used to sequence closely linked regions that have been used as proxies for the diversity at the MAT loci. For several species, the *mip* gene has been used as a proxy for MAT A (James 2007; James *et al.* 2004a; Kües *et al.* 2001).

The tetrapolar mating system provides a high outcrossing efficiency with the prevention of inbreeding (*i.e.*, mating of primary mycelia/spores from the same fruit body). In basidiomycete populations, it will be advantageous to bear rare MAT alleles because the probability of mating by encountering mycelia with different mating types then increases. A similar situation applies for the MHC system in animals and the SI system in plants (reviewed in Charlesworth *et al.* 2005; Edwards & Hedrick 1998). It is believed that this “rare allele advantage” is the mechanism behind the high MAT allele diversity in basidiomycetes. Especially in genetically depleted populations, there will be a strong selection for rare MAT alleles. Because very high numbers of MAT alleles typically are present in natural population of basidiomycetes (Casselton & Kües 2007; Raper *et al.* 1958), a population with generally low genetic diversity might be more suitable for analyzing the richness and distribution of MAT alleles.

The tetrapolar basidiomycete fungus *Serpula lacrymans* is an aggressive brown rot decayer, attacking wooden constructions in buildings in temperate regions worldwide. The species probably originated in mainland Asia, from where it has dispersed worldwide by the help of human activity (Kauserud *et al.* 2007). Various genetic markers have demonstrated that low levels of genetic variation occur in the European founder population (Engh *et al.* 2010a; Kauserud *et al.* 2007). Four different mating factors at one locus and five in the other were detected by Schmidt and Moreth-Kebernik (1991) in an *in vitro* crossing experiment between European strains. The same number of mating factors was confirmed present by Kauserud *et al.* (2006). In strong contrast to the low levels of variation observed at neutral loci in *S. lacrymans*, high levels of molecular variation was detected in a MAT A linked region (part of the *mip* gene, intergenic spacer and HD1) by Engh *et al.* (2010b). Strong negative frequency dependent selection acting on MAT A was suggested to maintain the high levels of molecular variation (Engh *et al.* 2010b); however, no direct (functional) link was made between the genotype and MAT alleles.

The primary aim of this study was to identify and investigate the distribution and richness of MAT alleles in *S. lacrymans* in Europe. We first annotated the mating type regions by using information from the newly sequenced genomes of *S. lacrymans* (Eastwood *et al.* 2011). We then selected two genetic markers, being linked to the two MAT loci, as representatives (proxies) for the MAT alleles. We tested the functionality of the MAT proxies by intrastock crossings of a spore family. Finally, we investigated the distribution and richness of MAT alleles in the European founder population using the developed markers.

## Material and Methods

### Mating type annotation

The mating type loci of the genome sequenced strain S7.9 was partially annotated on the JGI genome browser (http://genome.jgi.doe.gov/) (Eastwood *et al.* 2011). This information was used as a base for further annotations of the mating type loci of the S7.9 and S7.3 strains. The mating type loci were annotated based on similarity to other species where the mating type loci have been characterized, including *Laccaria bicolor*, *Schizophyllum commune*, *Pleurotus djamor*, *Coprinopsis cinerea*, and *Phanerochaete chrysosporium* (James *et al.* 2004a, 2011; Kües *et al.* 1994; Martin *et al.* 2008; Martinez *et al.* 2004; Niculita-Hirzel *et al.* 2008; Ohm *et al.* 2010). Because mating type gene order is largely conserved among basidiomycetes, positional homology can be used in the search for the mating type genes. In this study we searched for sequence identity of the *mip* gene as a starting point of identifying the MAT A region. The MAT B region is more variable among species, and the pheromone precursor genes are smaller and difficult to identify. However, a cluster of STE3-like pheromone receptor genes and pheromone precursor genes is the standard arrangement of the MAT B region (Niculita-Hirzel *et al.* 2008). This cluster is also often linked to a Pak-kinase gene (James 2007; James *et al.* 2006). In addition, the pheromones have certain common features that are used for identification. They are modified on both the N and the C-termini on the translated amino acid sequence, with a postulated N-terminal cleaving site and a CaaX motif (C, cystein; aa, two aliphalic amino acids; X is any aa amino acid) that is prenylating the C-terminus of the pheromone (Niculita-Hirzel *et al.* 2008; Raudaskoski & Kothe 2010). During the annotation work, the software Geneious Pro 5.4.6 (Biomatter, Auckland, New Zealand) was used.

### Primer design for mating type proxies

**Figure 1:**
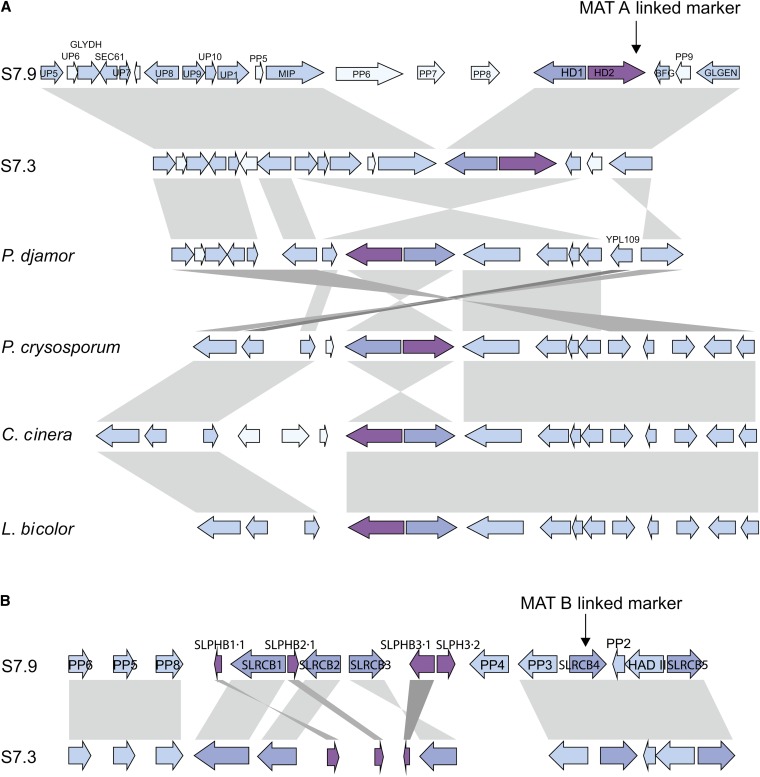
Annotation of the mating type loci for *Serpula lacrymans*, and synteny to other basidiomycete species. Arrows indicates genes and their transcribed direction. The gray shadings between strains/species indicate synteny. (A) Dark blue indicates HD1, dark purple indicates HD2, and blue indicates mating type linked genes with synteny in other species. Light blue indicates genes with no synteny among species. The black arrow indicates the region in which the primers for amplifying the MAT A linked marker is localized. (B) Purple indicates the pheromone receptor genes, dark purple indicate the pheromone receptor genes, and blue indicates mating type linked genes with synteny in the other strain. The black arrow indicates the region in which the primers for amplifying the MAT B linked marker is localized.

Adjacent regions to the MAT genes were selected as proxies to represent the mating types. Primers from MAT A and MAT B were designed based on the annotated genomes using the Primer3 software available as a plugin in Geneious Pro 5.4.6. In addition to the genome sequences of the S7.9 and S7.3 strains (Eastwood *et al.* 2011), unpublished reads from three Illumina sequenced 108-bp paired-end libraries of three other *S*. *lacrymans* strains were available. These reads were searched for the suggested primer sites, to ensure that two alleles could be identified for each strain. A marker for the MAT A region was located in the 3′UTR of the MAT A HD2 transcription factor (MATA11_F 5′-GCC TCT TGG TTG TTT TTA TTG 3′ and MATA7_R 5′-GCT GTG AGT GCT AGT GCT ACA-3′; Figure 1A). A marker for the MAT B region was located in the pheromone receptor gene 4 (Slrcb4; MATB1_F - 5′-TCC TTC GCA CCT CAT GGC AGC-3′ and MATB1_R - 5′-TCG TAG GAC GGC ATC CAA AGC-3′; Figure 1B).

### Culturing and isolation of a spore family

The strain *S. lacrymans* Lmsa110092 was isolated in 2010 from a fruit body developed between plaster-board and wood beams in the roof of a house situated in the region of Ile de France (France). The dikaryotic mycelium of Lmsa110092 was cultured on malt extract peptone agar (MEA) slants and maintained at 4° in the Brittany Culture Collection, France (http://www.univ-brest.fr/souchotheque).

*In vitro* fruiting of the strain Lmsa110092 was artificially induced by enclosing a 20-d-old culture in plastic sleeves and placed in the dark at 20°. After 10−12 d, fruit bodies were observed in the Petri dish. Monokaryotic strains of *S. lacrymans* were obtained by collecting basidiospores from these fruit bodies. The isolated basidiospores were suspended in sterile distilled water and Tween 80, and then diluted up to a solution of 10^3^ spores/mL. Then, 80 µL was spread onto potato dextrose agar plates that were sealed with Parafilm. The plates were incubated at 20° in the dark for 3 d to allow spores to swell. The larger swelled spores were easier to recognize and transfer individually. Using an inverted microscope (×100), we transferred 200 well-separated basidiospores individually to 3% MEA media by removing tiny agar plugs with a scalpel blade. The resulting monokaryotic cultures were microscopically checked for absence of clamp connections and 23 progenies derived from Lmsa110092 were included in this study.

### Mating experiments

We identified four strains of each of the four tentative mating types by sequencing the MAT A and MAT B proxies described previously. These 16 monokaryons were included for further analyses and crossed pairwise by placing inocula 2 cm apart on 10-cm Petri dishes containing 1% MEA media. Each cross was replicated twice, including self-crossings. The Petri dishes were incubated at 20° for 25 d and the confronted mycelia subsequently checked for clamp connection. However, because no dikaryotic strains without clamps are reported for *S*. *lacrymans* (Jennings & Bravery 1991), we considered two monokaryotic strains compatible if the crossings resulted in clamp formation.

### Samples included, DNA extraction, amplification, cloning, and sequencing

Genomic DNA was isolated from the 23 monokaryotic strains derived from Lmsa110092, three monokaryotic strains derived from S5 obtained from O. Schmidt’s culture collection, and 26 dikaryotic environmental strains (Table 1 and 2) growing on MEA media using the cetyltrimethylammonium bromide miniprep method as described by Murray and Thompson (1980). DNA of seven monokaryotic strains previously studied by Kauserud *et al.* (2006) also were included for the following steps (Table 2). A total of 25 µL of polymerase chain reaction (PCR) mixtures contain 3 µL of 10× DyNAzyme EXT buffer, 2.5 µL of dNTPs (2 mM stocks), 1.5 µL of each primer (5 µM stocks), 0.3 U of DyNAzyme EXT DNA polymerase, and 2 µL of DNA diluted around 30 ng/µL. Thermal cycling conditions consisted of an initial denaturation at 94° for 2 min and 30 cycles of 30 sec at 94°, 45 sec at 57° annealing for MAT A primers and 52° for MAT B primers, 72° extension for 1 min for MAT A and 30 s for MAT B, and a final 72° extension for 7 min.

**Table 1:** Strains of *Serpula lacrymans* included in this study, their locality and their genotype of the two mating type sequenced proxies

ID	Country	Locality	MAT A	MAT B
CZ2	Czech	Jihlava	A1, A2	B1, B3
Lmsa111007	France	Brest	A2, A3	B1
Lmsa110074	France	Lentillère	A1, A2	
Lmsa110076	France	Commana	A3	B3
Lmsa110092	France	Buc	A1, A3	B2, B3
SL2	Norway	Oslo		B4
SL3	Germany	Bad Bevensen	A2, A3	B1, B2
SL4	Germany	Rothenburg	A1, A3	B3 B4
SL87	Norway	Oslo	A1	B3, B4
SL146	Czech		A3	
SL160	Norway	Drammen	A1, A3	B2, B4
SL161	Norway	Haugesund	A3	B1, B3
SL164	Norway	Renneby	A1, A3	B1, B4
SL186	England	Hampton Hill	A1, A2, A3	B1, B2
SL200	Poland	Warsaw	A1, A3	
SL203	England	London	A1, A2	B2, B4
SL204	Scotland	Glasgow	A1, A2	B2, B4
SL219	Finland	Helsinki	A2, A3	B2, B4
SL220	Finland	Manlyharju	A2, A3	B1, B4
SL223	Finland	Pernio	A3	B2, B4
SL224	Finland	Myrskyla	A1, A2	B1, B3
SL234	Belgium	Gembloux	A1, A3	B1, B4
SL236	Belgium	Bruxelles	A1, A2, A3	B1, B2
SL286	Norway	Hønefoss	A1, A2, A3	B2, B3
SL290	Norway	Steinkjer	A1, A3	B2, B3
SL487	Poland	Warsaw	A1	

**Table 2:** Monokaryons used in previous mating studies (Kauserud *et al.* 2006; Schmidt and Moreth-Kebernik 1991) that are sequenced in this study

ID	Type A Factors	Type B Factors	MAT A	MAT B
S3.3	A1	B4	A2	B2
S3.9	A2	B3	A3	B3
S3.10	A1	B3	A2	B3
S7.3	A1	B1	A2	B4
S7.8	A1	B2	A2	B1
S7.9	A2	B2	A3	B1
S12.5	A1	B5	A2	B3
S5.2	A4	B4	A3	B2
S5.3	A4	B1	A3	B4
S5.13	A3	B4	A1	B2

Functional mating factors from previous mating experiments are indicated in columns 2 and 3, whereas the genotypes based on the mating type proxies from this study are indicated in columns 4 and 5

The PCR-amplified DNA products from dikaryotic strains were purified using the QIAquick PCR purification kit (QIAGEN) and were cloned into TOPO-TA vector (Invitrogen) following manufacturer’s instructions. For each PCR product, eight clones were directly amplified with the T7/M13R primers. All PCR products were purified for sequencing using ExoSAP-IT and run on a 3730 XL DNA analyzer (Life Technologies, Foster City, CA) at the ABI-lab at CEES, University of Oslo. The sequences were trimmed and aligned in Geneious Pro 5.6.4. Parsimony trees were made in TNT (Goloboff *et al.* 2008), and strict consensus trees are presented as simplified unrooted cladograms. The sequences from the MAT B linked marker have been accessioned in GenBank with the following accession no.: KC182670-KC182721. The sequences from the MAT A linked marker have been deposited in the Dryad repository: http://dx.doi.org/10.5061/dryad.gr6b8.

## Results

### Annotation of the MAT regions

A synteny map of the MAT A region of the two *S*. *lacrymans* strains and four other Agaricomycetes can be seen in Figure 1A. The MAT A locus of *S. lacrymans* includes one copy of each of the homeodomain transcription factors, HD1 and HD2. For the genome sequenced strain S7.9, these genes were located on Scaffold 1, position 5108560-5113562, and for strain S7.3 on Scaffold 21 position 162954-167421. HD1 and HD2 in S7.3 are, as for all investigated Agaricomycetes, flanked and closely linked by the mitochondrial intermediate peptidase (*mip*) and the beta-flanking gene (bfg; James 2007; James *et al.* 2004a; Kües *et al.* 2001). However, for S7.9 a 18-kb region has been inserted between *mip* and HD1. The inserted region contains three putative genes (PP6, PP7, and PP8, Figure 1A). One of these genes (PP8) had a significant sequence identity to a gypsy element when using the tBLASTx algorithm to search the Repbase (a database of transposable elements; Jurka *et al.* 2005). Gypsy elements are long-terminal repeats retrotransposons, found in most fungal species (Muszewska *et al.* 2011). Using the element to search the unmasked *S*. *lacrymans* genome, we found that the inserted region has several inverted repeats and that dispersed repetitive elements are found throughout the genome. This finding suggests that at least a part of the inserted region is a retrotransposon. The other putative genes (PP6 and PP7) in the retrotransposon are predicted proteins of unknown function, where homologous sequences of PP6 are found in many other Agaricomycetes. *Serpula lacrymans* lacks the gene YPL109 (a kinase of unknown function), which is present in all other species in the synteny map (Figure 1A). Otherwise, the gene organization is similar to many other species, in particular to that of *Pleurotus djamor* (Figure 1A) (James *et al.* 2004b; Martinez *et al.* 2009; Niculita-Hirzel *et al.* 2008).

The MAT B locus was identified by the combination of STE3-like pheromone receptor genes and pheromone precursor genes closely linked in a locus. For S7.9 five STE3-like pheromone receptor genes were found in combination with four pheromone genes in Scaffold 3 position 2697701-2719838 (Figure 1B), and Pak-kinase was found on the same scaffold in position 2739037-2742489. In S7.3, five STE3-like pheromone receptor genes were found, but only three pheromone genes are closely linked to the pheromone receptor genes in Scaffold 28 position 42257-66919. Pak-kinase was found on the same scaffold in position 20945-24397. Notably, the two MAT B alleles from S7.9 and S7.3 are inverted (not visible on Figure 1B). Two STE3-like pheromone receptor genes (Slrcb4 and Slrcb5) were identical in both strains (Figure 1B). These two genes did not have adjacent pheromone precursor genes and, hence, are probably not mating type pheromone receptor genes. Thus, it seems that *S. lacrymans* has one MAT B locus with three STE3 receptor genes and their compatible pheromones. One of the receptor genes has two adjacent pheromones for S7.9 (Slphb3.1 and Slphb3.2, Figure 1B, supporting information, Table S1).

### Distribution of MAT alleles in the *S*. *lacrymans* spore family

Twenty-three monokaryotic strains were obtained from the dikaryotic (parental) Lmsa110092 isolate. DNA sequences obtained from the two MAT linked markers from the 23 monokaryons indicated that all four combinations of MAT alleles were present with 5, 5, 6, and 7 monokaryons each. Four monokaryons of each combination were included in the *in vitro* crossings. The results from the crossings corresponded fully with the expectations; clamp connections were formed in the hetero-allelic combinations and vice versa (Table 3).

**Table 3:** Results from intra-stock crossings of a spore family of the strain Lmsa110092 of *Serpula lacrymans*.

		R1	R2	R7	R18	R4	R13	R19	R22	R12	R14	R20	R21	R9	R10	R16	R17
Isolate ID	Mating type	A1B3	A1B3	A1B3	A1B3	A3B2	A3B2	A3B2	A3B2	A1B2	A1B2	A1B2	A1B2	A3B3	A3B3	A3B3	A3B3
R1	A1B3		−	−	−	+	+	+	+	−	−	−	−	−	−	−	−
R2	A1B3			−	−	+	+	+	?	−	−	−	−	−	−	−	−
R7	A1B3				−	+	+	+	+	−	−	−	−	−	−	−	−
R18	A1B3					+	+	+	+	−	−	−	−	−	−	−	−
R4	A3B2						−	−	−	−	−	−	−	−	−	−	−
R13	A3B2							−	−	−	−	−	−	−	−	−	−
R19	A3B2								−	−	−	−	−	−	−	−	−
R22	A3B2									−	−	−	−	−	−	−	−
R12	A1B2										−	−	−	+	+	+	+
R14	A1B2											−	−	+	+	+	+
R20	A1B2												−	+	+	+	+
R21	A1B2													+	+	+	+
R9	A3B3														−	−	−
R10	A3B3															−	−
R16	A3B3																−
R17	A3B3																

+ indicates that dikaryotic mycelia with clamp connections are formed; − indicates lack of dikaryon formation; ? indicates one crossing experiment where the two monokaryons never met.

### Distribution of MAT alleles in European *S*. *lacrymans* strains

**Figure 2:**
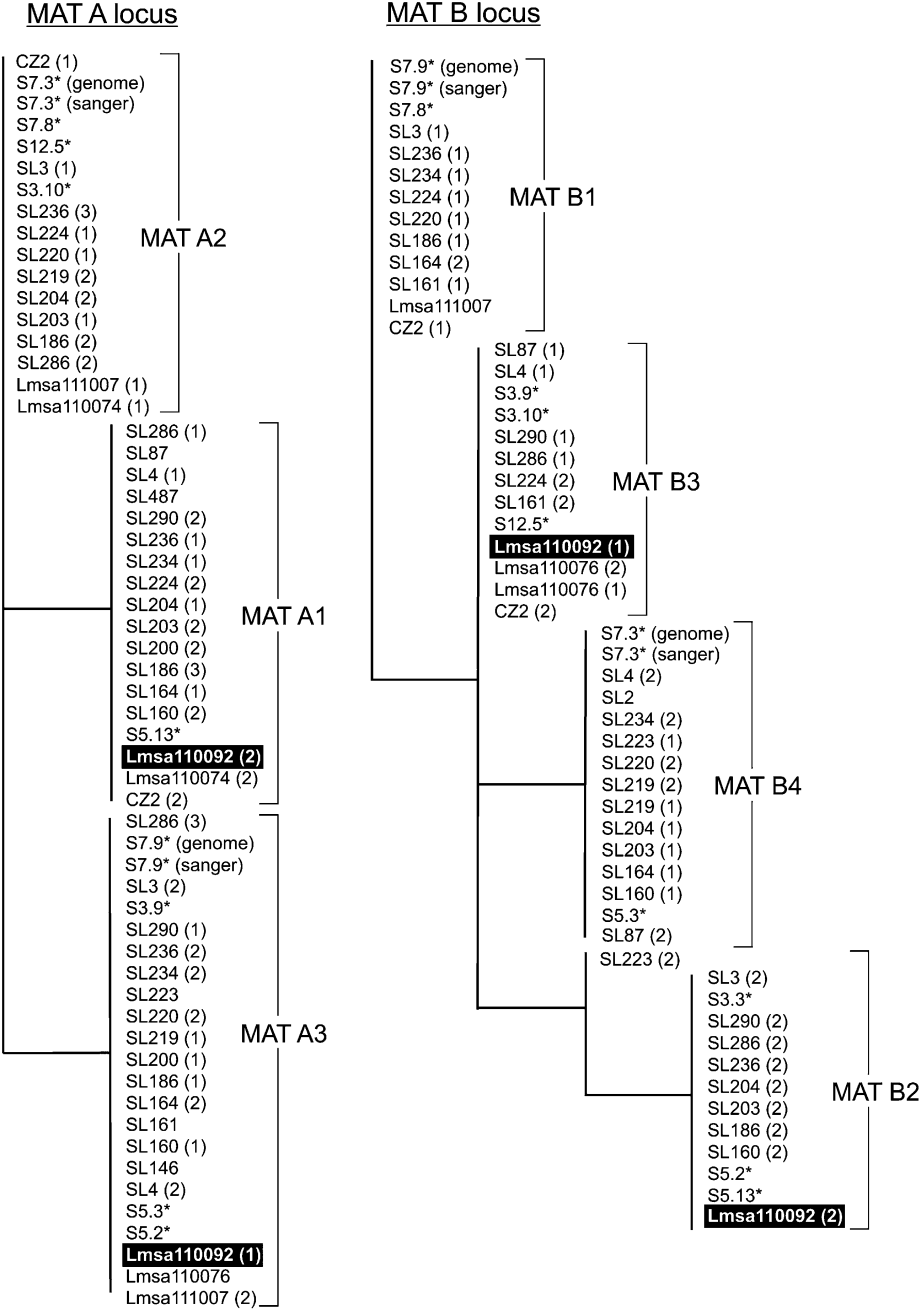
Cladograms of the sequenced mating type proxies for MAT A and MAT B for the European population of *Serpula lacrymans*. Asterisks indicate sequenced monokaryons, numbers in parentheses indicate the allelic identity of the cloned dikaryons, and Lmsa110092 with a black background indicate the dikaryon that was used for the *in vitro* intra-stock crossings.

Sequence analyses of the MAT linked markers revealed that three MAT A alleles tentatively are present in the 26 analyzed European mono- and dikaryons (Tables 1 and 2; Figure 2). These strains were distinguished by three equally distributed alleles (Table 1 and 2; Figure 2). Four MAT B alleles were found, equally distributed in the analyzed sample (Tables 1 and 2; Figure 2). When comparing the MAT linked marker sequences of the monokaryons previously investigated in Schmidt and Moreth-Kebernik (1991) and Kauserud *et al.* (2006), we found that functional mating factor A was indeed homologous to MAT A in other basidiomycetes and that the functional mating factor B was homologous to MAT B.

For some strains of the dikaryotic European population we found only one allele of either MAT A (6 strains) or MAT B (2 strains; Table 1). This could be due to too few sequenced colonies or that our mating type proxies could not discriminate among all functional mating types. The last hypothesis is supported by the fact that two functional mating factors from the previous studies was not distinguished by our mating type marker (Table 2; S12.5 and S5.3). For three dikaryotic strains we found three MAT A alleles. Whether this is due to contamination, presence of three nuclei or duplications of the loci is unknown

## Discussion

### Annotation and synteny of the MAT regions in *S*. *lacrymans*

Synteny, with only two inversions, was found between *S*. *lacrymans* and *P*. *djamor* in the MAT A region. Overall, there was a conserved gene organization among the species investigated. However, the retrotransposon found in S7.9 MAT A was surprising because this region has been suggested to be highly conserved in Agaricomycetes (James 2007; Niculita-Hirzel *et al.* 2008). The same retrotransposon was identified in other European dikaryotic strains with primers spanning *mip* and the retrotransposon (Engh *et al.* 2010b). The retrotransposon separated *mip* and *bfg* in S7.9 by approximately 24 kb. A retrotransposon also was found in both MAT A of *Postia placenta* and *Phanerochaete chrysosporium*, separating *mip* and *bfg* with about 20 and 18 kb, respectively (James *et al.* 2011; Martinez *et al.* 2009). In *P*. *chrysosporium* another insert in the region downstream of HD1 was found in two other individuals. This is in contrast to the expected homology among species for the MAT A flanking regions. The two putative proteins, PP6 and PP7, were probably inserted with the retrotransposon. Transposable elements have been suggested to be important in the formation of homothallic species by facilitating rearrangements of MAT loci (Gioti *et al.* 2012). In general, it is known that the level of repetitive DNA in sex chromosomes of animals and plants, and mating type loci in fungi, is higher than for other regions of the genome. This is explained by accumulation of repetitive DNA in low recombining sex linked regions of the genome (Fraser & Heitman 2005).

Lower levels of synteny of the gene organization were found within *S. lacrymans* for the MAT B locus (Figure 1B). Similarly, Niculita-Hirzel *et al.* (2008) found lower levels of synteny among the MAT B region, than for the MAT A region of several Agaricomycetes. They suggested the MAT B locus was subject to higher recombination rates than MAT A (Niculita-Hirzel *et al.* 2008). This was argued as the distance among genes in the MAT A region was shorter than the distances found in the MAT B region. In line with this, longer intergenic regions in MAT B than for MAT A were also found in our study.

### Linking MAT genotypes and phenotypes

A full consistency was found between the genotypes of the MAT linked markers and the results from the *in vitro* intra-stock crossings. This finding suggests that the developed markers largely represent the functional mating types and can be used to study the richness and distribution of MAT alleles in *S. lacrymans*. Our results indicate that for species where genome data are available, MAT linked markers can easily be developed and used for analyzing their mating systems, as previously suggested (James 2007; James *et al.* 2004a; Kües *et al.* 2001). Nevertheless, two functional mating types (B5 and A4 from Kauserud *et al.* 2006) was not recognized among the genotypes, which is not surprising as the MAT linked markers only functions as proxies for the whole MAT gene complexes. Thus, care has to be taken when concluding about the exact number of MAT alleles in the European population.

The presence of more than two alleles for some of the strains could theoretically be explained by three or more nuclei present in the mycelia. In our study we did not count the number of nuclei in each cell, but rather trusted the presence of clamp connections as evidence for dikaryotic mycelium. More than two nuclei has not been reported previously for *S*. *lacrymans* by nuclei counting, but Engh *et al.* (2010b) also found some strains with three copies of the *mip*-HD1 mating type proxy.

### Diversity of MAT alleles among European strains

Amplification of a part of the *mip* gene and the intergenic spacer between *mip* and HD1 in a large population of *Serpula* identified three main clades of European *mip* alleles (Engh *et al.* 2010b). These clades were congruent with the three MAT A alleles found in our study. In the MAT B linked marker, we found four alleles. Altogether, these numbers correspond largely with the number of MAT alleles encountered in earlier *in vitro* crossing based studies of European *S. lacrymans* monokaryons (Kauserud *et al.* 2006; Schmidt & Moreth-Kebernik 1991). The low number of MAT alleles in European *S. lacrymans* contrast the high number of alleles normally observed in natural basidiomycete populations. In a classic study by Raper *et al.* (1958), 339 MAT A mating types and 64 MAT B mating types were estimated in *Schizophyllum commune*. For *Coprinopsis cinerea* between 164–240 and 79–240 have been estimated for MAT A and MAT B, respectively (Casselton & Kües 2007; Day 1963; Kimura 1952). Our results support the hypothesis that *S. lacrymans* was introduced to Europe causing a bottleneck/founder event. In fact, the introduction of two dikaryons to Europe can account for the entire MAT allelic diversity in Europe. The low number of MAT alleles present in Europe might hinder a large proportion of potential dikaryon formations. Hence, the introduction of novel genetic material of *S. lacrymans* to Europe might lead to higher aggressiveness in the overall population. In the Dutch elm disease pathogen *Ophiostoma novo-ulmi*, a transfer of MAT and vegetative incompatibility alleles through rare interspecific crossings have been documented, which lead to increased levels of sexuality and genotypic diversity (Brasier *et al.* 1998).

### Can limited diversity of MAT alleles account for the observed heterozygote excess?

In previous population genetic studies of the European *S*. *lacrymans* population using microsatellite markers, heterozygote excess has been observed (Engh *et al.* 2010a,b). This was hypothesized to be either due to a recent bottleneck followed by genetic drift, or that the applied polymorphic microsatellite markers were linked to the regions under frequency dependent selection, including the MAT genes. However, comparison of the genomic locations of the employed microsatellite primers (Högberg *et al.* 2006) to the *S*. *lacrymans* genome revealed that the microsatellite loci were evenly distributed throughout the genome (data not shown). In theory, for genes subject to strong frequency-dependent selection, allelic richness may be maintained in the population following a bottleneck. However, even if the selection is strong, it is not expected that other loci than those tightly linked to genes under selection will experience elevated allelic diversity (Robertson 1962). In comparison, the major histocompatibility complex locus is fundamental to the vertebrate immune system, and is known to evolve under balancing selection (Aguilar *et al.* 2004; Gaudieri *et al.* 2000). High diversity of the major histocompatibility complex locus has been found in several organisms in which the diversity of neutral evolving loci was low, *e.g.*, in voles (Oliver & Piertney 2012) and foxes (Aguilar *et al.* 2004). This supports that the diversity of MAT alleles, as well as molecular variation in the linked DNA regions may be maintained in a population through a severe bottleneck, while neutral loci lose diversity.

## Conclusions

In this study we have, based on *S. lacrymans* genome data, developed MAT A and B linked markers that can be used for studying the allelic richness and distribution of MAT alleles in *S. lacrymans*. Moreover, through intrastock crossings of monokaryons with known marker genotypes, we corroborated a link between the MAT genotypes and phenotypes. The developed genetic markers did not recognize two of the functional mating types and, hence, probably lack some resolution. Despite this, the markers can be used further to analyze the diversity of MAT alleles in other *S. lacrymans* populations, including the natural population in Northeast Asia where a much higher allelic diversity is expected.

## Supplementary Material

Supporting Information
