# Supplementary material for: Molecular Characterization of Sexual Diversity in a Population of Serpula lacrymans, a Tetrapolar Basidiomycete
Source: G3 (Bethesda). 2013 Feb 1;3(2):145–52. doi: 10.1534/g3.112.003731 (PMC3564976; doi:10.1534/g3.112.003731)
Supplement: Supporting Information [file supp_3_2_145__index.html]

Supporting Information 

# Molecular Characterization of Sexual Diversity in a Population of *Serpula lacrymans*, a Tetrapolar Basidiomycete

## Supporting Information for Skrede, Maurice, and Kauserud, 2013

**Files in this Data Supplement:**

- Table S1 - Amino acid sequences of putative pheromone precursor genes for *Serpula lacrymans* S7.9 and S7.3 (PDF, 82 KB)
