# Supplementary material for: Molecular Characterization of Sexual Diversity in a Population of Serpula lacrymans, a Tetrapolar Basidiomycete
Source: G3 (Bethesda). 2013 Feb 1;3(2):145–52. doi: 10.1534/g3.112.003731 (PMC3564976; doi:10.1534/g3.112.003731)
Supplement: Supporting Information [file supp_3.2.145_003731SI.pdf]

**Molecular characterization of sexual diversity in a population of *Serpula lacrymans*, a tetrapolar basidiomycete**

Inger Skrede[§*1], Sundy Maurice[§,#] , Håvard Kauserud[§]

[§]Microbial Evolution Research Group (MERG), Department of Biology, University of Oslo, N-0316 Oslo, Norway
[#]Université de Brest, UEB, Laboratoire Universitaire de Biodiversité et Ecologie Microbienne, 29280 Plouzané, France

[1]Corresponding author: Microbial Evolution Research Group (MERG), Department of Biology, University of Oslo, PO Box 1066 Blindern, N-0316 Oslo, Norway

DOI: 10.1534/g3.112.003731

**Table S1 Amino acid sequences of putative pheromone precursor genes for *Serpula lacrymans* S7.9 and S7.3.** Bold indicates the postulated N-terminal cleavage site. Italics indicates the CaaX motif which is modified by C-terminal prenylation

| Gene ID | Strain | Amino acid sequence |
|---|---|---|
| Slphb1.1 | S.7.9 | MDSFTTLDLTIHPSETVHVEQDPASIPVNE**DT**PWRPGTY*CVIA**** |
| Slphb2.1 | S.7.9 | MDSFATISIQDNSSYLDVDIESSIPVNE**DS**PWRPGTY*CVIA**** |
| Slphb3.1 | S.7.9 | MDSFTVIEFTTTHQITTTDSPSLTTSQSEDASVMVNF**EH**DNNGSSGVGWF*CVIA**** |
| Slphb3.2 | S.7.9 | MDSFTTFELIAPDAPIFPSSEDDQVLIDA**DT**KAGYAGY*CVIT**** |
| Slphb1.1 | S7.3 | MDTFSSILDTFTPEVSQPSDEEFSPVNT**EY**PSLGGQSGF*CVIA**** |
| Slphb2.1 | S7.3 | MDSFTTISLAALSVESSETSTQFSSVACEAKSVWDAESPPVDS**DN**LSGYYGSF*CVIA**** |
| Slphb3.1 | S7.3 | MDTFTTLQSLSSDESATVSALPIATSVEEVYAIPMEF**EH**QGSGGPGWF*CTIA**** |
